# Low-Temperature Electron Spin Resonance Study of MnPS_3_ Antiferromagnetic Single Crystal

**DOI:** 10.1021/acs.jpcc.4c06156

**Published:** 2024-10-25

**Authors:** Fabrizio Moro, Bing Wu, Iva Plutnarová, Jan Plutnar, Zdenek Sofer

**Affiliations:** † Department of Materials Science, University of Milano-Bicocca, via R. Cozzi 55, Milano 20125, Italy; ‡ Department of Inorganic Chemistry, Faculty of Chemical Technology, University of Chemistry and Technology Prague, Technická 5, 166 28 Prague 6, Czech Republic

## Abstract

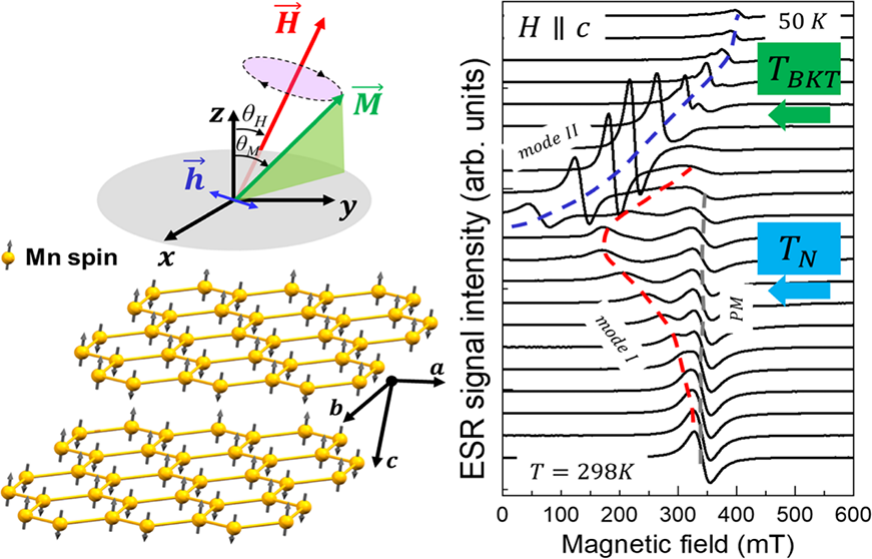

van der Waals MnPS_3_ compound belonging to the class of Néel-type antiferromagnets (AFM) has recently emerged as a promising two-dimensional material for spintronic applications. In this study, we report on the electron spin resonance (ESR) study of an MnPS_3_ single crystal across the Néel transition temperature (*T*_N_ = 78 K) for the magnetic field applied in the directions parallel and perpendicular to the crystallographic *c* axis. Furthermore, the ESR angular dependence at a temperature near *T*_N_ has been investigated. We observed multiple resonance modes with antiferromagnetic, ferromagnetic, and paramagnetic characters. In addition, we revealed the occurrence of complex spin–spin correlations and a magnetic-topological Berezinskii–Kosterlitz–Thouless (BKT) phase transition (i.e., bound vortex-antivortex pairs) at *T*_BKT_ with *T*_BKT_/*T*_N_ typically found in two-dimensional magnets.

## 1 Introduction

Layered van der Waals magnets, also referred to as 2D magnets, are gaining more and more interest because of their rich physical phenomena and potential applications in spintronics.^1,2^ Particularly appealing is the possibility to form heterostructures from mechanical exfoliation of bulk crystals down to few layers or even the monolayer. These heterostructures would be functional to realize magnetic tunneling junctions for spin-transfer torque (STT), spin–orbit torque (SOT), and thermally assisted (TA) magnetic random-access memories (MRAM), which are considered the only nonvolatile memories with high density, endurance, and fast writing speed.^3,4^

The most widely studied 2D magnets are those with a ferromagnetic (FM) ground state,^5^ whereas research on antiferromagnetic (AFM) 2D magnets has not proceeded at the same pace mainly because of the difficulties in detecting detailed magnetic properties in materials with weak or null magnetization in the ground state. Nevertheless, several recent experiments have confirmed the possibility to store magnetic information with AFM materials.^6−10^ With respect to FM materials, AFM-based devices provide the advantage of avoiding unintentional magnetic *crosstalk* between neighboring devices because of zero stray fields. Thus, AFM 2D magnets would enable magnetic storage robust against magnetic field perturbations.^7^

Among the class of AFM 2D magnets, the MPS_3_ (M = Fe, Ni, and Mn) family stands out because magnetic order, spin dimensionality, and transition temperature depend on M despite alike crystal structures. FePS_3_ and NiPS_3_ show zigzag-like AFM ordering, whereas MnPS_3_ shows a Néel-type AFM configuration where the spin of each magnetic site is antialigned with the two nearest neighbors.^11−13^ In the ordered state, FePS_3_ and MnPS_3_ show an easy axis perpendicular to the crystallographic plane, whereas NiPS_3_ possesses an easy plane coinciding with the atomic planes.^14^ Furthermore, the bulk Néel temperatures for FePS_3_,^15−17^ NiPS_3_,^18^ and MnPS_3_,^14^ are 118 K, 155 and 78 K, respectively.^19^

Despite the lower Néel temperature, MnPS_3_ is particularly attractive because bulk-like magnetism might persist down to the monolayer limit,^20^ as well as because of interesting phenomena such as induced ferromagnetism^21^ and spin flop transition^13^ at low temperature in the presence of magnetic field. Recently, the possibility to observe a magnetic-topological Berezinskii–Kosterlitz–Thouless (BKT) phase transition, which stabilizes a bound vortex–antivortex pair at BKT temperature, *T*_BKT_, has been suggested.^22,23^

Nevertheless, a distinction must be made between type-A layered AFMs, which possess ferromagnetic coupling within the layers, and Néel-layered AFMs in which spins are arranged antiferromagnetically within each layer. In the former, magnetic tunneling junctions can be realized by changing the relative orientation between the layers. In the latter, spin-filtering effects do not occur and the magnetic properties cannot be monitored with conventional transport measurements.^20^ Therefore, the magnetic characterization of Néel-layered AFMs requires alternative techniques. Electron spin resonance (ESR) and the related electron paramagnetic resonance (EPR), antiferromagnetic resonance (AMR), and ferromagnetic resonance (FMR) techniques have been proved to be powerful tools to study 2D magnets.^24^ Temperature magnetic resonance field, linewidth, and intensity enable the characterization of the magnetic transition phases, whereas the angular dependence of the magnetic resonances in a single crystal provides unambiguous determination of the magnetic axes. Recently, ESR has shown to be a valid tool to assess the spin dimensionality and find signatures for magnetic topological phase transitions of 2D magnets in the bulk 3D form from combined angular and temperature dependences of the spectral linewidth supported by theoretical modeling.^25,26^ This is possible because 2D magnets possess very weak interlayer magnetic coupling relative to the in-plane coupling, which makes them inherently 2D even in their bulk form.

Previous ESR studies on MnPS_3_ have been reported by a few groups at conventional X-band frequency^23^ as well as at frequencies in the 8–330 GHz frequency range.^22,27^ The high frequencies/strong magnetic fields studies have reported the occurrence of AFM and paramagnetic resonance branches^22,27^ as well as the occurrence of a transition from 3D-like character of the spin wave excitation to a 2D-XY scaling regime boosted by the strong magnetic field.^22^ Whereas, at X-band, only a single broad line has been reported in the temperature region above the Néel temperature (*T* > 100 K).^23,28,29^

Here, we investigate the temperature and angular dependence of the ESR spectra on an MnPS_3_ single crystal in the relevant temperature range crossing *T*_N_ from room temperature to liquid helium temperature. Our ESR spectra show the occurrence of previously unreported resonance branches above the transition temperature *T*_N_ = 78 K, revealing a complex magnetic behavior of MnPS_3_ with mixed ferromagnetic and antiferromagnetic phases, which could be responsible for the formation of magnetic topological phases. Finally, we discuss and interpret the ESR based on the BKT model of the ESR linewidth.

## 2 Methods

### 2.1 Crystal Synthesis

MnPS_3_ was made by direct reaction in ampule from elements with subsequent chemical vapor transport crystal growth.^30,31^ Manganese (99.95%, −100 mesh, Mateck, Germany), phosphorus (99.9999%, 2–6 mm granules, Wuhan Xinrong New Materials Co., China), and sulfur (99.9999%, 2–6 mm granules, Wuhan Xinrong New Materials Co., China) were placed in a quartz ampule (50 × 250 mm) in a stochiometric ratio corresponding to 30 g of MnPS_3_. To facilitate chemical vapor transport, sulfur and phosphorus were used in 1 at % excess together with 0.5 g of iodine (99.9%, Fisher Scientific, USA). The ampule was sealed under high vacuum using oxygen–hydrogen welding torch and diffusion pump with liquid nitrogen trap (pressure <1 × 10^–3^ Pa). The ampule was placed in a muffle furnace and heated at 450 °C for 50 h and subsequently in steps at 500 °C for 50 h, 600 °C for 50 h, and 700 °C for 50 h. The heating rate between each step was 0.2 °C/min. Subsequently, the ampule was placed in a two-zone furnace for crystal growth. First, the growth zone was heated at 750 °C and the source zone at 500 °C. After 2 days, the gradient was reversed, and the source zone was heated at 750 °C, while the growth zone temperature was decreased from 700 to 650 °C over a period of 5 days, and for the following 5 days, the growth zone was kept at 650 °C. At the end of crystal growth procedure, the growth zone was heated at 350 °C, while the source zone was kept at 150 °C for 2 h. The ampule was opened in an argon-filled glovebox, where crystals with size over 1 cm were separated. The resulting bulk MnPS_3_ crystals are air stable and do not need any protection layer. For the measurement, no protection layer was needed and crystals could be handled on air. Exfoliated layers are more susceptible to degradation by air humidity and should be handled on air only for a short time. In ambient atmosphere, MnPS_3_ can be safely handled up to at least 100 °C.

### 2.2 X-ray Diffraction (XRD)

The crystal structure was characterized using an X-ray diffractometer (XRD, Bruker D8 with Cu Kα radiation, Germany) and Raman analysis (using a 532 nm laser, Renishaw, England), and transmission electron microscopy (TEM, EFTEM Jeol 2200 FS microscope, Japan).

### 2.3 X-ray Photoelectron Spectroscopy (XPS)

The surface composition of the samples was further studied with X-ray photoelectron spectroscopy (XPS) using a SPECS spectrometer equipped with a monochromatic Al Kα X-ray source (1486.7 eV) and a hemispherical electron analyzer Phoibos 150. The survey spectra were recorded with energy E_p_ set to 100 eV and the high-resolution spectra of the core lines with E_p_ set to 20 eV. The base chamber pressure during the acquisitions was at 10^–9^ mbar or lower. Due to a heavy charge development on the surfaces of samples, a low-energy electron flow generated by an electron flood gun has been used to compensate the charge buildup.

### 2.4 Transmission Electron Microscopy (TEM)

TEM, including high-resolution TEM and SAED, was recorded on an EFTEM Jeol 2200 FS microscope, Japan. Energy-dispersive X-ray (EDX) analysis was also conducted using a detector from Oxford Instruments in the same instrument.

### 2.5 Raman analysis

The Raman resonance spectra were recorded using a confocal Raman microscope (Renishaw, UK) with a DPSS Nd:YAG laser (532 nm), 20× lens, 0.5 mW on sample (1%), 20 s exposure time, and 30 scans.

### 2.6 Electron Spin Resonance

Samples were mounted on a Suprasil quartz rod and into a goniometer with 1/2 degree sensitivity for ESR crystal rotation measurements. A flat crystal with surface size 2 × 3 mm^2^ and thickness <1 mm was selected to avoid saturation and distortion of the ESR signal. The ESR spectra were recorded with a Varian E15 spectrometer coupled to a Bruker super high Q cavity operated at X-band (∼9.4 GHz). The static magnetic field was modulated at 100 kHz with a modulation amplitude of 4 G. The magnetic field was continuously monitored with an electronic counter and a Hall probe. Sample temperatures in the range 4–300 K were obtained with an Oxford ESR 900 cryostat. A DPPH sample was used as a reference to determine the *g*-factors. MatLab-generated routines were used to fit the spectra. Peak-to-peak linewidths Δ*H* were calculated from the full-width half-maximum Δ*H*_1/2_ obtained from the Lorentzian fit with the relation: Δ*H =* Δ*H*_1/2_/.

## 3 Results

**Figure 1:**
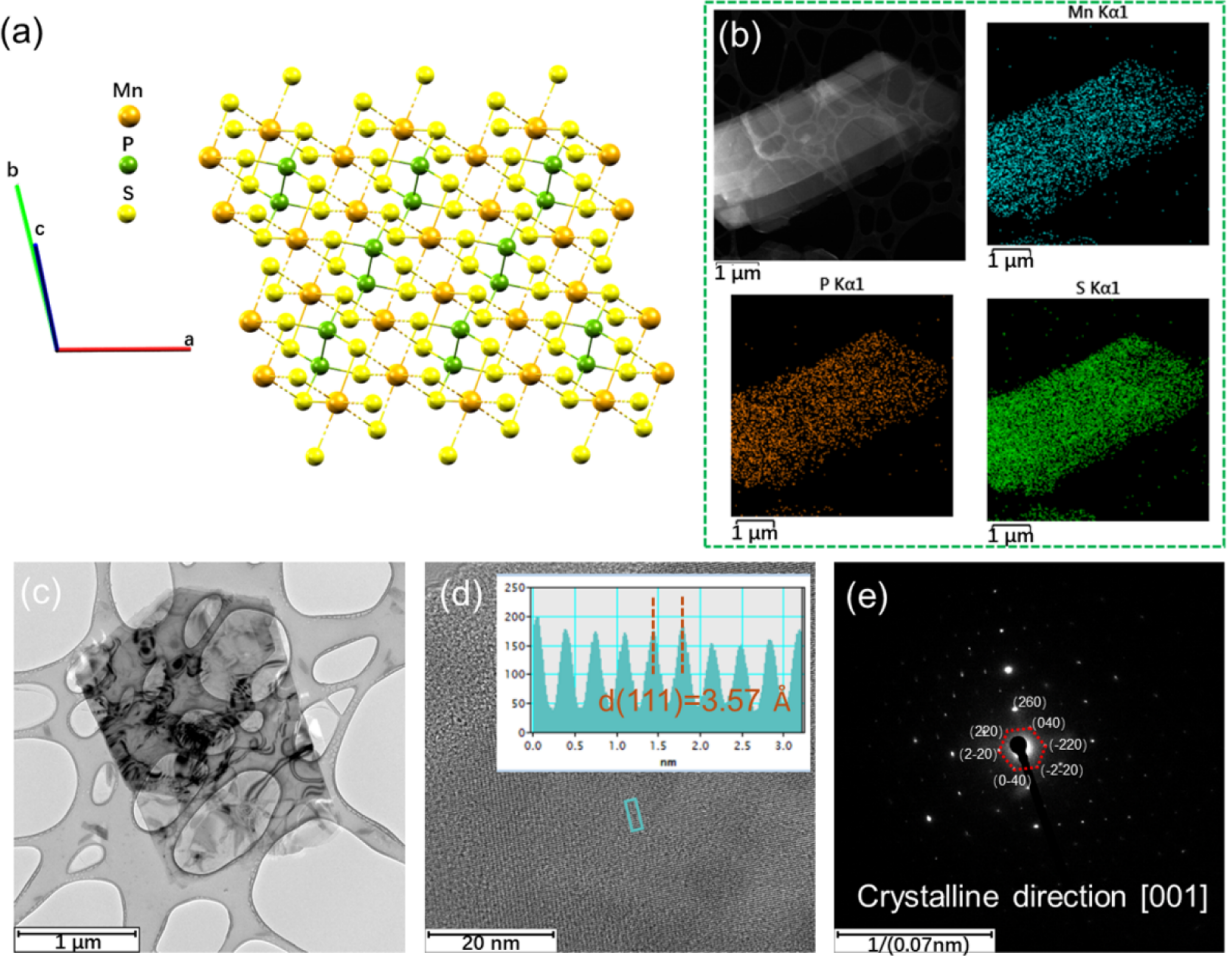
(a) Crystal structure of MnPS_3_. (b) EDS mapping indicating the elemental distribution of Mn, P, and S within MnPS_3_. (c) TEM image and corresponding high-resolution TEM image with an inset showing the lattice fringe spacing. (d) SAED pattern and (e) the [001] zone axis of MnPS_3_.

The X-ray diffraction (XRD) structure of MnPS_3_ is shown in Figure 1a. The diffraction pattern presented in Figure S1 indicates high crystalline quality and a well-ordered structure of the MnPS_3_ single crystal with *C*2*/m* space group. EDS mapping images shown in Figure 1b provide a spatial distribution of manganese (Mn), phosphorus (P), and sulfur (S) within the sample, confirming the homogeneous composition and elemental distribution across the crystal. The corresponding EDS spectrum of MnPS_3_ in Figure S2 shows the ratio of Mn:P:S in weight is 14.8%:8.4%:25.6%, corresponding to the atomic ratio of 1.0:1.01:2.96, matching well with the designed stoichiometric ratio of the compound’s formula. High-resolution TEM (HRTEM) reveals the atomic lattice with a measured lattice spacing (d) of 3.57 Å for the (111) planes, consistent with known crystallographic data for MnPS_3_ (Figure 1c,d). The SAED pattern displays sharp and well-defined diffraction spots, confirming the crystal direction as [001] and indicating high crystallinity and structural integrity (Figure 1e). These results are also supported by additional XPS and Raman studies reported in Figures S3 and S4.

**Figure 2:**
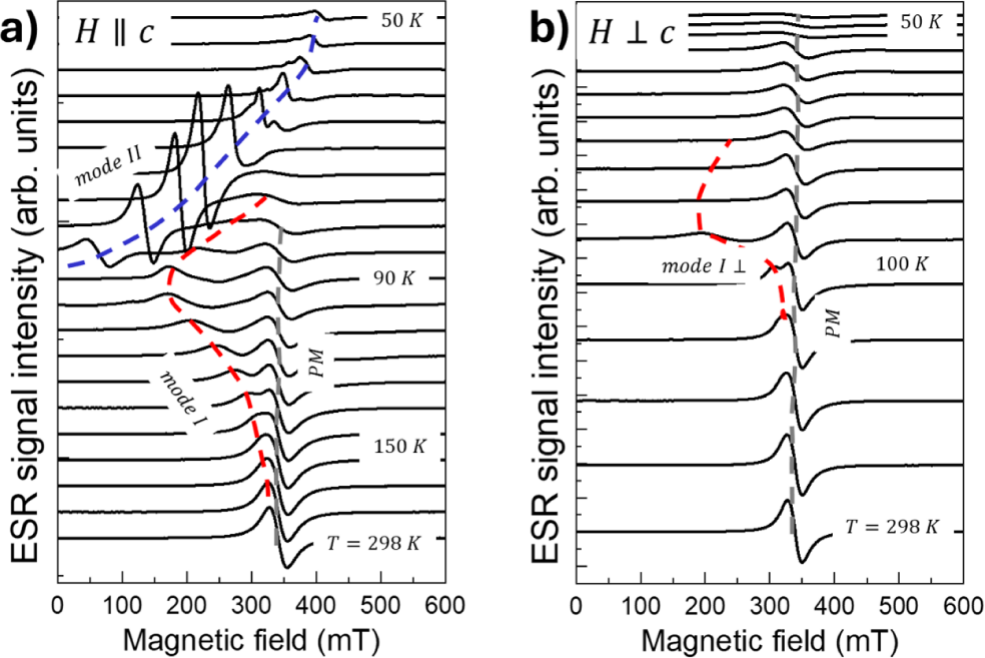
ESR spectra recorded as a function of the temperature for *H*||*c* (a) and *H*⊥*c* (b). The dashed lines are guide for the eyes to single out the different resonance lines: PM, *mode I*, and *mode II*.

Figure 2 shows the temperature dependence of the ESR spectra recorded for the external magnetic field parallel (*H*||*C*) and perpendicular (*H*⊥*c*) to the crystallographic *c* axis. In both cases, there is a single ESR peak centered at *H*_*r*_ = 342 *mT* and *H*_*r*_ = 339 *mT*, respectively, whose resonance fields are essentially independent of temperature. We refer to the central resonance peak as PM due to its paramagnetic behavior, as discussed further. Upon cooling for *H*||*c* we observe the emergence of a secondary resonance branch that overlaps with the PM signal at *T* ∼ 150 K, and it evolves down to*T* ∼ 50 K. Hereafter, we refer to this signal as *mode I*. For *T* < 94 K another resonance signal, *mode II*, appears from very low magnetic field values.

For *H*⊥*c,* only one resonance signal is observed down to *T* ∼ 50 K with a weak contribution from *mode I* appearing for *T* ∼ 100 K.

We extracted the ESR intensity, resonance peak (*H*_r_), and peak-to-peak linewidth (Δ*H*) as a function of temperature and rotation for *H*||*c* and *H*⊥*c* from fitting of the spectra to Lorentzian functions.

**Figure 3:**
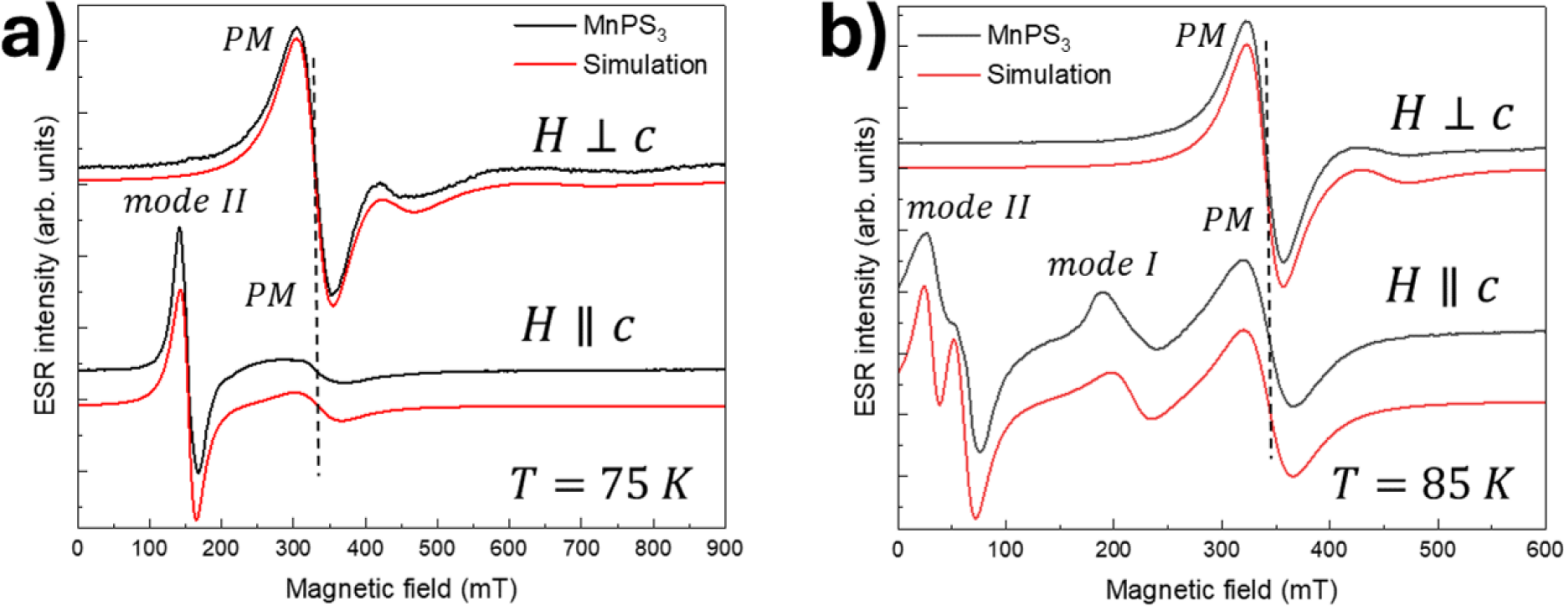
ESR spectra recorded for *H*⊥*c* and *H*||*c* for *T* ∼ 75 K (a) and *T* ∼ 85 K (b). Black curves are experimental data, and red curves are the simulations. Spectra are offset along the vertical axes for clarity.

In Figure 3 are reported stack plots of the simulation and the experimental data for the canonical orientations and for two representative temperatures, namely, *T* = 75 K and *T* = 85 K.

**Figure 4:**
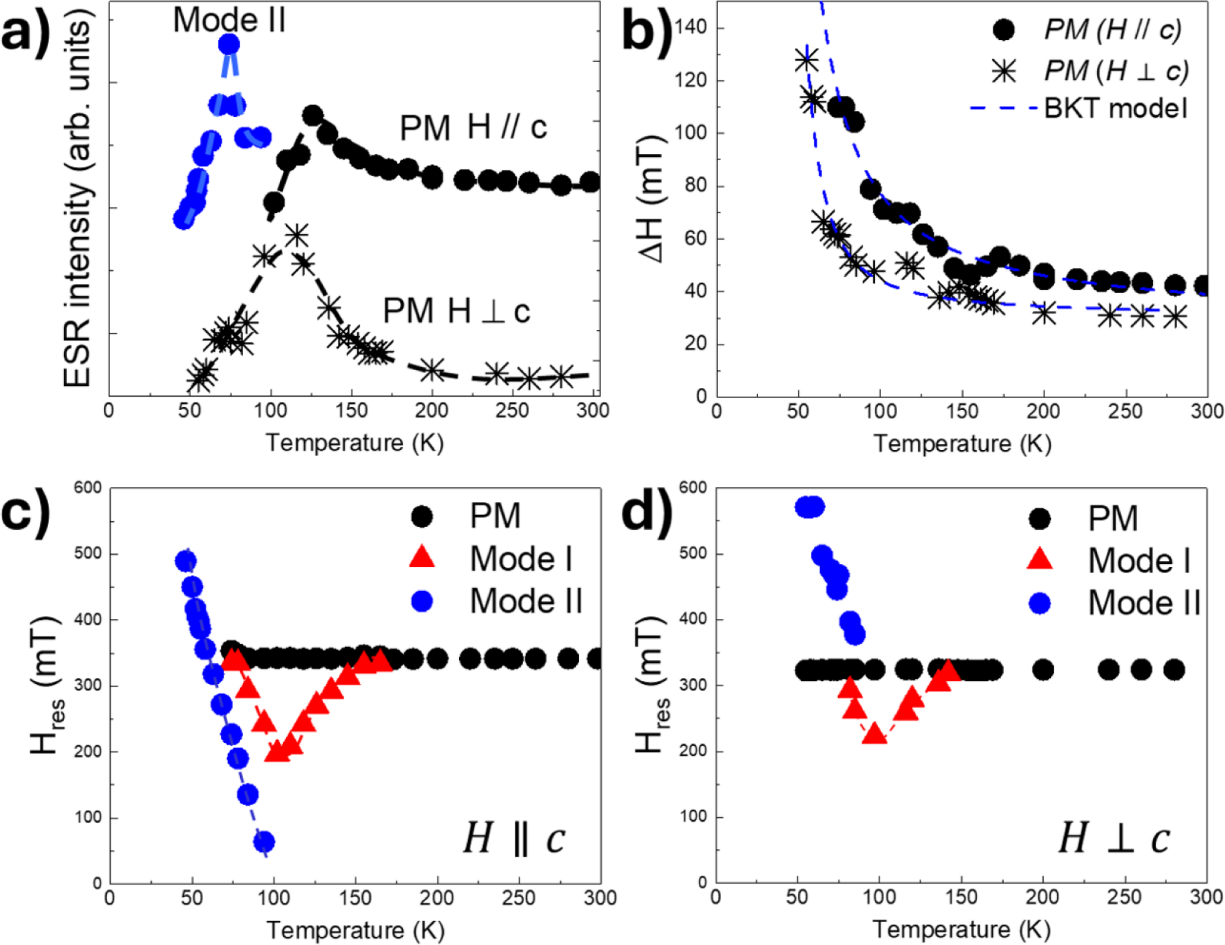
Temperature dependence of the ESR intensity (a), peak-to-peak linewidth (b) for the PM and mode II resonances for *H*⊥*c* and *H*||*c*. In (b), dashed lines are fittings of the data to the BKT model. Resonance fields are for *H*⊥*c* (c) and *H*||*c* (d). Line are guides for the eyes.

We now describe in detail the temperature dependence of PM and *mode I* and *mode II* reported in Figure 4. *PM* intensity for *H*||*c* increases by lowering the temperature to *T* ∼ 130 K before decreasing at lower temperatures. *Mode I* intensity decreases monotonously from room temperature to *T* ∼ 80 K. For *T <* 100 K, the *mode II* emerges with a rapidly increasing intensity, reaching a maximum at *T* ∼ 74 K before quickly decreasing at lower temperatures. PM intensity for *H*⊥*c* increases by lowering the temperature down to *T* ∼ 120 K before decreasing at lower temperatures and vanishing for *T <* 100 K.

The resonance field for PM is essentially independent of temperature for both orientations *H* ||*c* and *H*⊥*c*, whereas its resonance linewidth Δ*H* increases monotonously upon cooling until *T* ∼ 150 K, and at lower temperatures, it rapidly broadens for *T <* 100 K. Resonance field for *mode I* for both *H*⊥*c* and *H*||*c* separates from PM for *T <* 150 K and shifts to lower fields, reaching a minimum for *T* ∼ 100 K before shifting to higher resonance fields. The resonance field of *mode II* for *H*||*c* appears from the zero field at *T <* 100 K and shifts linearly to higher magnetic fields.

**Figure 5:**
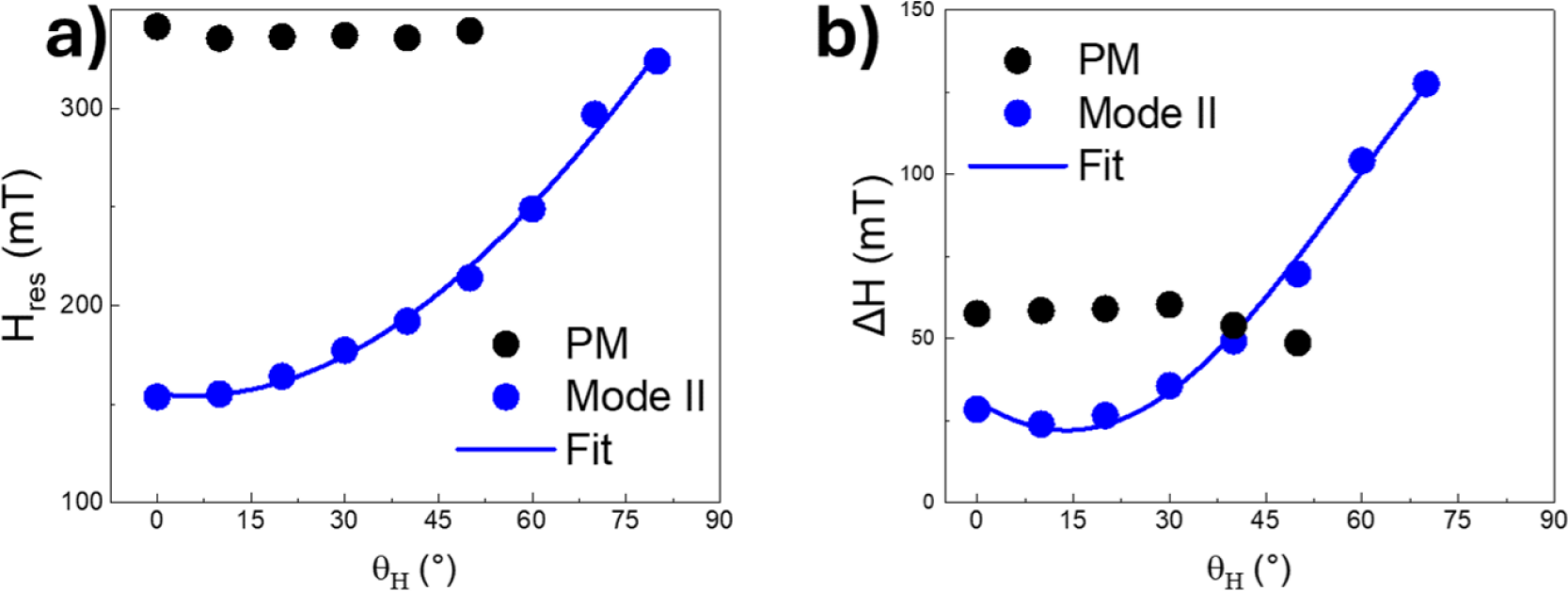
Angular dependence of the resonance field (a) and ESR linewidth (b) for the PM and *mode II* ESR signals for *T* = 75 K.

The angular dependence of the resonance field, *H*_r_, and peak-to-peak linewidth, Δ*H*, obtained from the best fit at *T* = 75 K for PM and *mode II* are reported in Figure 5. *H*_r_ and Δ*H* for the PM signal depend weakly on the orientation of the crystal with respect to the magnetic field, whereas a pronounced angular dependence is observed for *mode II. H*_r_ and Δ*H* show a minimum for *H*||*c* and a maximum for *H*⊥*c*.

## 4 Discussion

The observed PM signal is associated with a paramagnetic phase in the MnPS_3_ crystal because it is isotropic and its resonance field is essentially temperature independent. This interpretation is also supported by the analysis of its ESR intensity, which increases for lower temperatures in agreement with the Curie’s law for paramagnets. The decrease in the ESR intensity for *T* < 150 K indicates the onset of an antiferromagnetic phase, where the antiparallel alignment of the Mn spins effectively decreases the magnetic susceptibility. The shift of *mode I* to lower and higher fields in the temperature region 150–90 K (Figure 4c,d) suggests the occurrence of mixed antiferromagnetic and ferromagnetic phases. The last one is supported by *mode II,* which appears from zero magnetic field at *T* ∼ 90 K, corresponding to the temperature upturn of *mode I*. For *T* < 90 K *mode II* shifts quickly to high fields at lower temperatures, thus suggesting its AFM nature further corroborated by the quenching of its ESR intensity. These results elucidate the complex mixing of AFM and FM where the degree of mixing might give rise to different spin arrangements.

The van der Waals nature of MnPS_3_ crystals suggests that magnetic order could be confined in a 2-dimensional space and described by the XY spin model below the magnetic phase transition. Within such a model, topological effects can lead to the formation of local spin arrangements such as bound vortex and antivortex pairs, as predicted by the BKT theory. The ESR spectra can capture the signature of the BKT transition in both the temperature and angular dependence of the ESR linewidth. The ESR linewidth follows the temperature dependence according to the BKT model:^32^
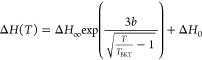
1where in the first term, Δ*H*_∞_ is the linewidth to infinity, *b* = π/2, and Δ*H*_0_ takes into account an offset of the room temperature linewidth (Table 1).

**Table 1:** Results of the Fitting to eq 1

BKT model	Δ*H*_∞_ (mT)	b	Δ*H*_0_ (mT)	*T*_BKT_ (K)
*H*||*c*	4 ± 1	π /2	183 ± 10	28 ± 1
*H*⊥*c*	17 ± 5	π /2	170 ± 20	25 ± 3

From the fitting of the data in Figure 3b, we find a substantial agreement between the *T*_BKT_ obtained for both orientations within the fitting error bars. The value of *T*_BKT_ is always below the magnetic ordering temperature *T*_N_, with a ratio , as typically found in quasi-two-dimensional magnets.^26^

The angular dependence of the resonance field and linewidth of the PM resonance below *T* ∼ 90 K is very weak, confirming its isotropic character in the entire temperature range investigated. A pronounced ESR angular dependence is observed for *mode II*. In order to search for the formation of bound vortex and antivortex pairs, we fit the ESR resonance linewidth angular dependence of *mode II* at *T* = 75 K. Experimental evidence indicates that the ESR linewidth in the regime of mixed FM and AFM phases, which is responsible for the formation of topological phases, deviates from a simple *cos*^2^φ dependence:
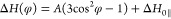
2a

Instead, it assumes what it is usually referred to as a *W*-like shape, which can be fitted with the following equation:
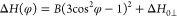
2b

The fitting of the ESR linewidth to eq 2a suggests the occurrence of only one dominant magnetic phase (i.e., FM), whereas the ESR linewidth at *T* = 75 K clearly deviates from a cos^2^φ-like shape leaning more toward a *W*-like shape. This argument is supported by the fitting of the data to eq 2b with parameters: *B =* 153 ± 7 mT and Δ*H*_0⊥_ = 22 ± 6 *mT*.

## 5 Conclusion

We have reported on a temperature and angular study of an MnPS_3_ single crystal by ESR at conventional X-band frequency. We have observed the occurrence of a paramagnetic phase along with multiple resonance modes above and below the Néel temperature for both the magnetic field applied along and perpendicular to the crystallographic *c* axis. We have ascribed these modes to competing AFM and FM interactions, as suggested by their opposite shift of the resonance fields. From the analysis of the resonance linewidth, we have obtained evidence for spin–spin correlations at *T* < *T*_N_, which we ascribe to the magnetic topological phase transition and to the formation of bound vortex and antivortex pairs of the BKT model.

## Data Availability

The datasets generated during and/or analyzed during the study are accessible via the Zenodo repository: https://zenodo.org/records/13692266 and Bicocca Open Archive Research Data, doi: 10.17632/x7zhjtzy5s.1.

## Supporting Information Available

XRD studies, EDS spectrum of MnPS_3_, XPS characterization, and Raman studies (PDF)
