# Supplementary material for: Low-Temperature Electron Spin Resonance Study of MnPS3 Antiferromagnetic Single Crystal
Source: J Phys Chem C Nanomater Interfaces. 2024 Oct 25;128(45):19306–12. doi: 10.1021/acs.jpcc.4c06156 (PMC11573119; doi:10.1021/acs.jpcc.4c06156)
Supplement: Supplementary file 1 — jp4c06156_si_001.pdf [file jp4c06156_si_001.pdf]

SUPPLEMENTARY INFORMATION

Low Temperature Electron Spin Resonance Study of $MnPS_3$ Antiferromagnetic Single Crystal

Fabrizio Moro[a*], Bing Wu[b], Iva Plutnarová[b], Jan Plutnar[b], Zdenek Sofer[b*]

[a] *Department of Materials Science, University of Milano-Bicocca, via R. Cozzi 55, Milano 20125, Italy.*

[b] *Department of Inorganic Chemistry, Faculty of Chemical Technology, University of Chemistry and Technology Prague, Technická 5, 16628 Prague 6, Czech Republic*

* Email: fabrizio.moro@unimib.it

* Email: zdenek.sofer@seznam.cz

Keywords: $MnPS_3$; 2D magnets; electron spin resonance; BKT transition

S1. XRD studies

The diffraction pattern of $MnPS_3$ presented in **Figure S1** reveals several sharp and intense diffraction peaks corresponding to different crystallographic planes, specifically (001), (002), (003), (004), and (005). These reflections indicate the high crystalline quality of the $MnPS_3$ single crystal with *C*2/*m* space group. The well-defined peaks confirm the layered structure of $MnPS_3$, typical for this type of material, with narrow peak widths and high intensity signifying excellent crystallinity and a well-ordered structure. The absence of additional peaks suggests that the $MnPS_3$ single crystal is free from impurities and secondary phases.

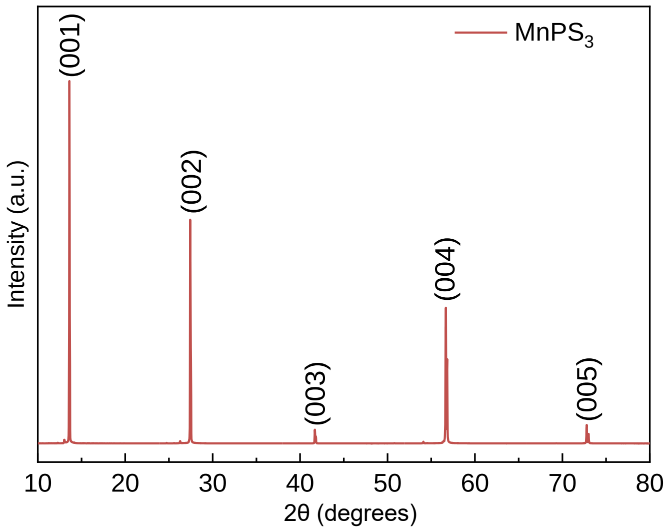

Figure S1 X-ray diffraction pattern of $MnPS_3$.

S2. EDS spectrum of $MnPS_3$

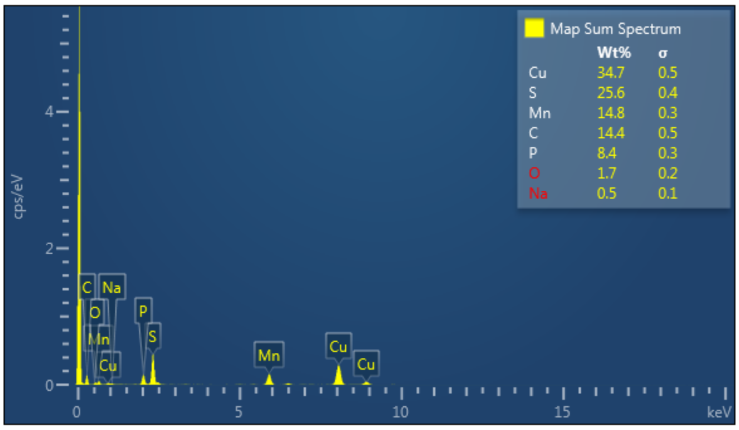

Figure S2 EDS spectrum of $MnPS_3$ crystal.

S3. XPS characterization

The XPS spectra of $MnPS_3$ are shown in **Figure S3**. The spectrum of the core $2p$ electrons exhibits the expected pair of regions attributable to the $2p_{3/2}$ and $2p_{1/2}$ spin-orbit interaction. Each of these two regions contains two broad signals, with intensity maxima in the $2p_{3/2}$ region at 639.2 and

643.7 eV, and in the $2p_{1/2}$ region at 650.9 and 656.7 eV, respectively. The intensity ratio of these two signals is approximately 37:63 and the signals are assigned to the two possible final electron configurations of the photoionized Mn atom - $2p^5 3d^6 \underline{L}$ and $2p^5 3d^5$, respectively. [1] The lower binding energy signal could be fitted with three bands corresponding to the splitting of the energy states of the high-spin material, as described by Gupta et al. [2] The spectrum of the core 3*s* electrons shows the presence of two broad signals at 84.5 and 89.9 eV, respectively, corresponding to the ^7S and ^5S final states possible for the $3s^1 3d^5$ electron configuration of the photoionized atom. The measured energy splitting of these two states (Δ = 5.4 eV) is comparable to the splitting reported for MnS (5.3 eV)[3] and corresponds to a relatively strong covalent character of the Mn-S bonds. In the 3*p* level region, there are three main bands present with maxima at binding energies approximately 48.8, 52.3, and 68.0 eV, corresponding to the ^7P and ^5P final states of the 3*p* photoionized atom. [4]

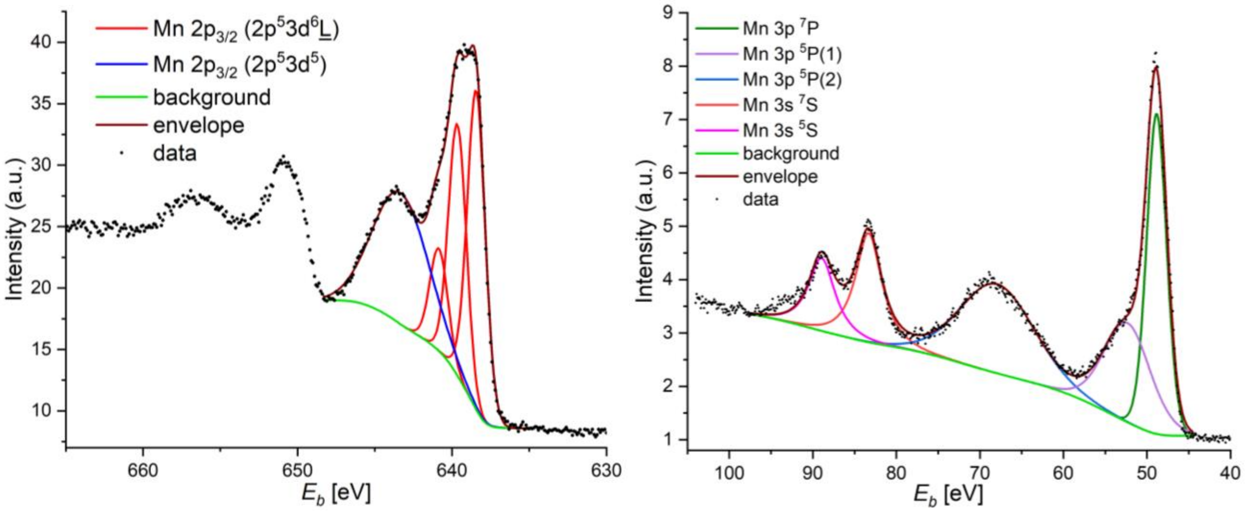

Figure S3 The XPS spectra of the Mn 2p (left), 3s and 3p (right) core lines of $MnPS_3$.

Table S1 reports the extracted integrated areas of the Mn 2p, P 2p and S 2p peaks. As it can be seen from the the calculated atomic percentage analysis, the stochiometric ratio is in a reasonable agreement with the expected 1:1: 3 ratio.

Name	Binding Energy (eV)	FWHM (eV)	Area (a.u.)	At %
Mn 2p	640	3.48	1102547.95	18.35
P 2p	132	3.73	146797.94	18.79
S 2p	162	3.16	690870.65	62.86

Table S1 Binding energy, Full Width at Half Maximum (FWHM), Area and At% for the XPS peaks corresponding to Mn2p, P2p and S2p.

S4 Raman studies

The room temperature Raman resonance spectrum in the range below 700 cm^{-1} is shown in **Figure S4**. The spectrum exhibits eight resolved lines of different intensity. The most intense bands at 384.4 and 274.3 cm^{-1} correspond to the symmetric stretching mode and bending mode of the PS_3 groups. The two bands present in the higher-energy part of the spectrum – the lines at 569.6 and 582.2 cm^{-1} correspond to the weakly degenerate P-P stretching mode of the P_2S_6 groups. The remaining four bands in the low energy part of the spectrum at 245.2, 225.2, 155.8, and 116.8 cm^{-1} correspond to the chalcogen in-plane displacement and metal in-lane displacement modes, respectively.[5]

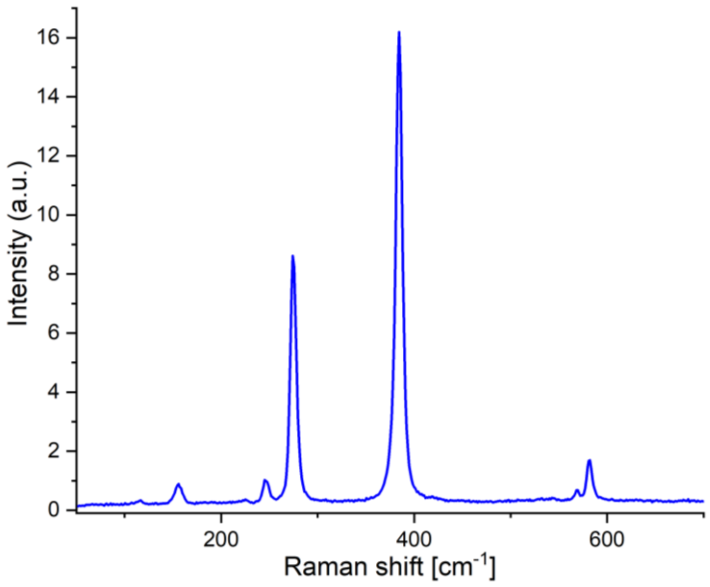

Figure S4 The room temperature Raman spectrum of a $MnPS_3$ crystal.

References

(1) Groot, F. d.; A., K. *Core level spectroscopy of solids*; CRC Press, 2008. DOI: 10.1201/9781420008425.

(2) Gupta, R. P.; Sen, S. K. Calculation of multiplet structure of core p vacancy levels. *Phys. Rev. B* **1974**, *10* (1), 71-77.

(3) Carver, J. C.; Carlson, T. A.; Schweitzer, G. K. Use of X-ray photoelectron spectroscopy to study bonding in Cr, Mn, Fe, and Co compounds *J. Chem. Phys.* **1972**, *57* (2), 973-+.

(4) Kowalczyk, S. P.; Ley, L.; McFeely, F. R.; Shirley, D. A. Multiplet splittng of manganese 2p and 3p levels in MnF_2 single crystals *Phys. Rev. B* **1975**, *11* (4), 1721-1727.

(5) Bernasconi, M.; Marra, G. L.; Benedek, G.; Miglio, L.; Balkanski, M.; Scagliotti, M.; Julien, C.; Jouanne, M. Lattice-dyanmics of layered MPX_3 (M=Mn, Fe, Ni, Zn, X=S, Se) compunds. *Phys. Rev. B* **1988**, *38* (17), 12089-12099.
